# *Arabidopsis* Basic Helix-Loop-Helix 34 (bHLH34) Is Involved in Glucose Signaling through Binding to a GAGA *Cis*-Element

**DOI:** 10.3389/fpls.2017.02100

**Published:** 2017-12-11

**Authors:** Ji-Hee Min, Hyun-Woo Ju, Dayoung Yoon, Kyeong-Hwan Lee, Sungbeom Lee, Cheol S. Kim

**Affiliations:** ^1^Department of Plant Biotechnology, Chonnam National University, Gwangju, South Korea; ^2^Department of Rural and Biosystems Engineering, Agricultural Robotics and Automation Research Center, Chonnam National University, Gwangju, South Korea; ^3^Korea Atomic Energy Research Institute, Daejeon, South Korea

**Keywords:** activator, AtPGR, bHLH34, glucose-responsive element, transcription factor

## Abstract

The modulation of glucose (Glc) homeostasis and signaling is crucial for plant growth and development. Nevertheless, the molecular signaling mechanism by which a plant senses a cellular Glc level and coordinates the expression of Glc-responsive genes is still incompletely understood. Previous studies have shown that *Arabidopsis thaliana* plasma membrane Glc-responsive regulator (AtPGR) is a component of the Glc-responsive pathway. Here, we demonstrated that a transcription factor bHLH34 binds to 5′-GAGA-3′ element of the promoter region of *AtPGR in vitro*, and activates beta-glucuronidase (GUS) activity upon Glc treatment in *AtPGR* promoter-GUS transgenic plants. Gain- and loss-of-function analyses suggested that the bHLH34 involved in the responses to not only Glc, but also abscisic acid (ABA) and salinity. These results suggest that bHLH34 functions as a transcription factor in the Glc-mediated stress responsive pathway as well as an activator of *AtPGR* transcription. Furthermore, genetic experiments revealed that in Glc response, the functions of bHLH34 are different from that of a bHLH104, a homolog of bHLH34. Collectively, our findings indicate that bHLH34 is a positive regulator of Glc, and may affect ABA or salinity response, whereas bHLH104 is a negative regulator and epistatic to bHLH34 in the Glc response.

## Introduction

Sugar signaling plays pivotal roles in modulating many features of germination, metabolism, growth, and development throughout the whole plant life cycle. Glucose (Glc), one of the hexose hydrolytic products of sucrose, is a major sugar signaling metabolite. Three Glc signal transduction pathways in plants have been suggested (Xiao et al., 2000). These are *Arabidopsis* *thaliana* hexokinase 1 (AtHXK1)-dependent pathway, in which photosynthetic gene expression correlates with the AtHXK1-mediated signaling function. The second one is a glycolysis-dependent pathway that requires AtHXK1 catalytic activity and regulates the expression of the pathogenesis-related (PR) genes (Xiao et al., 2000). The third pathway is involved in the regulation of a restricted number of genes such as those coding for cell wall invertase and chalcone synthase, and is independent of increased AtHXK1 activity (Xiao et al., 2000). Genetic evidence also indicates that a HXK-independent Glc-sensing and signaling mechanism involved in a G protein-coupled receptor system exists in plants (Chen and Jones, 2004).

Multiple Glc signal transduction pathways are intimately linked to developmental stages, hormones, and environmental conditions (Xiao et al., 2000; Eckardt, 2002). Glc signaling mutants have revealed a relation between Glc and abscisic acid (ABA) signaling pathways (Laby et al., 2000; Price et al., 2003). The ABA signaling transcriptional regulator ABA insensitive 4 (ABI4) represses the promoter of the ribulose-1,5-bisphosphate carboxylase (*RBCS*) gene in response to Glc or ABA (Acevedo-Hernández et al., 2005). Moreover, ABI4 is a regulator of mannose-induced inhibition of seedling germination (Pego et al., 1999), indicating a general role of ABI4 in hexose signaling. Several groups also proposed that the sucrose non-fermenting 1 (SNF1)-related protein kinase 1 (SnRK1) recognizes stress-associated energy or sugar limitation and promotes plant stress tolerance (Polge and Thomas, 2006). Also, SnRK1 is inactivated by Type 2C protein phosphatases, known repressors of the ABA pathway, and provided with evidences for the molecular connection between sugar and ABA signaling (Rodrigues et al., 2013).

A number of *cis*-regulatory elements responsible for the sugar-mediated gene modulation have been identified (Rolland et al., 2006), including G-box (Giuliano et al., 1988), W-box (Pla et al., 1993), SP8 motif (Ishiguro and Nakamura, 1994), sugar-response element (SURE) (Grierson et al., 1994), GC-box (Lu et al., 1998), osamy element (Lu et al., 2002), and the GCCT element (Chung et al., 2016). Recently, we reported that the *A. thaliana* Storekeeper-like 1 and 2 (AtSTKL1 and AtSTKL2) bind to the GCCT element in the *AtPGR* promoter region and suppress the gene activation (Chung et al., 2016). Overexpression of *AtPGR* modulates the induction of Glc and 2-deoxyglucose insensitivity under stress. In contrast, cotyledon greening of mutant seeds with an *atpgr* RNAi knockdown shows increased sensitivity to Glc and 2-deoxyglucose (Chung et al., 2011). AtSTKL homologs are capable of modulating the Glc response through the alteration of *AtPGR* expression. *AtSTKL*-overexpressing transgenic plants enhance the sensitivity to Glc in comparison with the wild type (WT), whereas *atstkl*s antisense plants show decreased sensitivity even with the high levels of Glc during cotyledon greening, indicating that AtSTKL play a significant role in Glc or Glc-mediated signaling pathways in *Arabidopsis* (Chung et al., 2016).

In the present study, we demonstrated that a transcription factor bHLH34 interacts with the 5′-GAGA-3′ *cis*-regulatory elements *in vitro* and activates the transcription of *AtPGR in planta*. bHLH34 was shown to act as a putative signaling protein in Glc, ABA, and high-salinity responses. The *bHLH34*-overexpressing transgenic plants showed enhanced resistance to Glc, ABA, and high salinity, whereas a decrease in *bHLH34* expression reduced Glc, ABA, and salt stress resistance at the early seedling stage. In addition, the expression changes of the stress-associated genes in *bHLH34* transgenic plants were consistent with the functions suggested by the physiological phenotype under abiotic stress conditions. Taken together, these results suggest that bHLH34 potentially functions as a factor linking Glc signaling and an abiotic stress response. Furthermore, genetic analyses suggested that bHLH34 has a function different from that of its highly homologous protein, bHLH104, in the Glc response.

## Materials and Methods

### Production of Recombinant bHLH34 Protein

For the DNA-binding analysis of the bHLH34 protein, full-length cDNA fragments were amplified using the following primers: for *bHLH34*, forward 5′-GGGGACAAGTTTGTACAAAAAAGCAGGCTTCATGTATCCATCAATCGAAGACGA-3′ and reverse 5′-GGGGACCACTTTGTACAAGAAAGCTGGGTCAGCAACAGGAGGAAGATTTTTGA-3′. The DNA products were introduced into the pDONR/ZEO vector (Invitrogen, Carlsbad, CA, United States) for DNA sequence analysis. The DNA fragment was then cloned into a pET300/NT-DEST vector by the Gateway system according to the manufacturer’s instruction (Invitrogen). The recombinant protein was expressed in the *Escherichia coli* strain BL21 (DE3) codon^+^ (Stratagene, La Jolla, CA, United States), and purified by ion exchange chromatography using Ni-charged His-bind resin (Novagen, Darmstadt, Germany).

### The Electrophoretic Mobility Shift Assay (EMSA)

**FIGURE 1:**
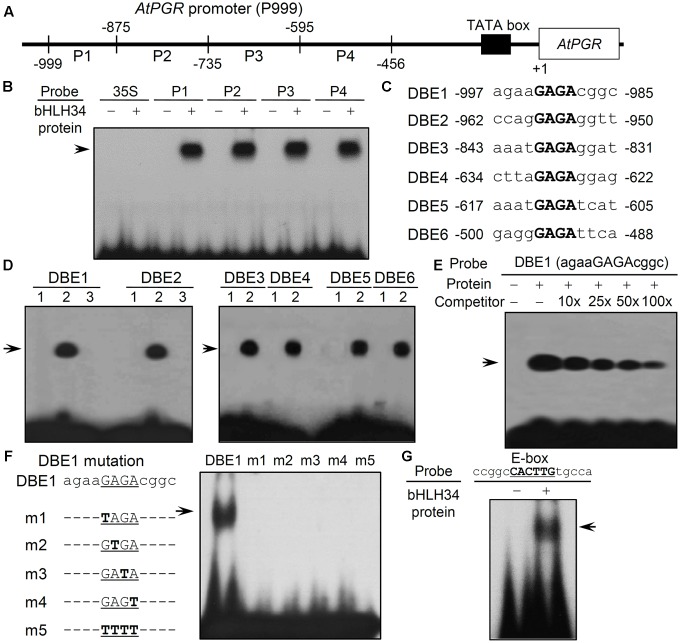
Electrophoretic mobility shift assay (EMSA) analyses of bHLH34. **(A)** A schematic diagram of the four (P1, P2, P3, and P4) fragments derived from the promoter 5′ upstream region (P999) of the *AtPGR* gene. The numbers indicate the nucleotide positions relative to the translation start site, ATG (A as +1). **(B)** Identification of the DNA-binding promoter fragment of the bHLH34 protein. Experiments were performed three times and similar results were obtained. The ^32^P-radiolabeled P1, P2, P3, and P4 DNA fragments incubated in the absence (–) or presence (+) of His-bHLH34 (arrow). 35S, 35S promoter without GAGA motif as a negative control. **(C)** Sequences of the six DNA binding elements (DBEs) in *AtPGR* promoter. GAGA motifs are shown in bold. **(D)** Identification of the DNA-binding promoter site in the bHLH34 protein. Experiments were carried out two times and similar results were obtained. Lane 1, the ^32^P-radiolabeled DBE1-6 oligonucleotides incubated in the absence of His-bHLH34; lane 2, the ^32^P-radiolabeled DBE1-6 oligonucleotides incubated in the presence of His-bHLH34 (arrow); and lane 3, the ^32^P-radiolabeled DBE1 or the ^32^P-radiolabeled DBE2 oligonucleotide incubated in the presence of MBP. **(E)** bHLH34 directly binds to the DBE1 oligonucleotide in the EMSA. The His-bHLH34 protein incubated with ^32^P-radiolabeled DBE1. An unlabeled DBE1 probe was used as the competitor (10-, 25-, 50- or 100-fold excess) to show binding specificity. The arrow indicates the shifted band. **(F)** bHLH34 specifically binds to the GAGA element. Mutations in GAGA element abolish bHLH34 binding to DBE1. A list of DBE1 and m1 to m4 point-mutated or four-nucleotide-mutated m5 probes. Mutated nucleotides are boldfaced. The binding sequence of bHLH34 was determined using various mutated sequences. The arrow indicates the position of the DBE1 probe-His-bHLH34 complex. **(G)** bHLH34 binds to E-box (ccggcCACTTGtgcca) in an EMSA. Lane 1, the ^32^P-radiolabeled- E-box (ccggcCACTTGtgcca) incubated in the absence of His-bHLH34; lane 2, the ^32^P-radiolabeled- E-box (ccggcCACTTGtgcca) incubated with His-bHLH34.

Four DNA fragments derived from the 5′-upstream region of *AtPGR* [see **Figure 1A**; P1, between nucleotide (nt) positions -999 and -875 relative to the *AtPGR* start codon site; P2, -874 to -735 nt; P3, -734 to -595 nt; P4, -594 to -456 nt] were synthesized by PCR [initial denaturation at 94°C for 2 min, amplification for 30 cycles (denaturation at 94°C for 15 s, annealing 58°C for 15 s, and extension at 72°C for 20 s) and final extension at 72°C for 3 min] with 8 primers (Supplementary Table S1). Six DNA binding elements (DBEs), including DBE1 (-997 to -985 nt), DBE2 (-962 to -950 nt), DBE3 (-843 to -831 nt), DBE4 (-643 to -622 nt), DBE5 (-617 to -605 nt), and DBE6 (-500 to -488 nt), and various mutated DBE1 oligonucleotides were prepared using the two complementary sequences were synthesized as shown in Supplementary Table S1. The double-stranded complementary oligonucleotides used as probes were radiolabeled with γ-^32^P-ATP and T4 polynucleotide kinase (New England BioLabs).

DNA–protein binding reactions were carried out at 25°C for 20 min in 20 μL volume containing 100 ng of each purified His-bHLH34 fusion protein, 1 μg of poly(dI-dC), 0.3 pmol of DNA fragments end-labeled with γ-^32^P-ATP, and DNA-binding buffer (10 mM Tris-HCl, pH 7.5, 2.5% glycerol, 50 mM KCl, 1 mM EDTA, and 1 mM DTT). The reaction mixture was subjected to 5% non-denaturing PAGE in TBE (44.5 mM Tris-hydroxymethyl aminomethane, 44.5 mM boric acid, and 1 mM EDTA) at 100 V for 2 h at 4°C. The gel with DNA–protein complexes was dried and X-ray film was exposed to this gel.

### Plant Materials, Growth Conditions, and Stress Induction

*Arabidopsis* seedlings were grown in a growth room under intense light (110 μmol m^-2^ sec^-1^) at 22°C, 60% relative humidity, and 16 h/8 h light/dark conditions. The bHLH104 T-DNA insertion line SALK_005802 (*bhlh104*) was acquired from the *Arabidopsis* T-DNA insertion collection of the Salk Institute (Alonso et al., 2003). To select plants homozygous for the T-DNA insertion, the gene-specific primers 5′-CTCCAGAAAGCGGTGAGTTTTG-3′ and 5′-GAAGAATCACAAGTTTCTGGAG-3′ (forward and reverse, respectively) were utilized for the *bhlh104* mutant. Plants yielding no PCR products with the gene-specific primers were subsequently tested for the presence of the T-DNA insertion using the gene-specific forward primer in combination with the T-DNA left border specific primer 5′-GCGTGGACCGCTGCACCT-3′.

The *AtPGR* promoter 999 (P999)-GUS construct was generated as described previously (Chung et al., 2016), and the overexpression of *bHLH34* in the P999-GUS transgenic lines was achieved as described in Supplementary Materials and Methods.

For Glc treatment, 14-day-old *Arabidopsis* seedlings were submerged in sterilized water containing 6% Glc and sampled at 0, 6, 12, and 36 h under continuous light condition (110 μmol m^-2^ sec^-1^ light intensity, 24 h light condition) at 22°C, and 60% relative humidity conditions. For ABA or salt stress, 14-day-old *Arabidopsis* seedlings were submerged in the sterilized water containing 100 μM ABA or 150 mM NaCl and sampled at 0, 3, 6, and 12 h. For osmotic stress, 14-day-old *Arabidopsis* seedlings were submerged in the sterilized water containing 400 mM mannitol and sampled at 0, 6, 12, and 36 h. In each case, the collected seedlings were promptly frozen in liquid nitrogen and stored at -80°C.

### Total RNA Extraction and qPCR Analysis

Total RNA was isolated from the frozen samples using the Plant RNeasy Extraction Kit (Qiagen, Valencia, CA, United States). To remove residual genomic DNA from the preparation, total RNA samples were treated with RNase-free DNase I in accordance with the manufacturer’s instructions (Qiagen). The concentration of RNA was exactly quantified via spectrophotometric measurements, and 3 μg of total RNA was separated on 1.2% formaldehyde agarose gels to verify its concentration and monitor its integrity. qPCR was performed on a Rotor-Gene 6000 quantitative PCR apparatus (Corbett Research, Mortlake, NSW, Australia), and each result was analyzed using the RG6000 1.7 software (Corbett Research). Total RNA samples were isolated from the variously treated 14-day-old seedlings using an RNeasy Plant Mini Kit (Qiagen). qPCR was conducted using the SensiMix One-Step Kit (Quantance, London, United Kingdom). *Actin 1* (*ACT1*) served as an internal control, and the quantitative analyses were performed by the Delta Delta *C*_T_ method (Livak and Schmittgen, 2001). Each sample was subjected to three independent experiments. RT-PCR was employed to measure the levels of *bHLH34* expression in transgenic plants. The amount of RNA used in RT-PCR reactions was 300 ng. After 28 PCR cycles of amplification, 20 μL of each RT-PCR product was loaded onto a 1.2% (w/v) agarose gel in order to visualize the amplified DNA. The primers used for qPCR and RT-PCR reactions are shown in Supplementary Table S2.

### Phenotypic Analysis and Stress Assays

For the Glc cotyledon greening test, seeds were sown on the Murashige and Skoog (MS) medium (Murashige and Skoog, 1962) supplemented with 5% or 6% Glc, and grown in a growth chamber under intense light (110 μmol m^-2^ sec^-1^) at 22°C, and 16 h/8 h light/dark conditions. Cotyledon greening was defined when a cotyledon was fully expanded and turned green. The green cotyledon was counted in triplicate (50 seeds/experiment).

For the ABA cotyledon greening or germination rate tests, seeds were sown on the MS medium supplemented with 0 or 1 μM ABA, and allowed to grow in the growth chamber under the same conditions as the Glc cotyledon greening assay. Germination was defined as an obvious protrusion of the root radicle through the seed coat. The germination rate of each line was measured after 1–8 days. Experiments were carried out in triplicate for each line (50 seeds each). Cotyledon greening of each seedling was measured at 14 days. The experiments were conducted in triplicate for each line (50 seeds each).

For the salt stress test, seeds were sown on the MS medium supplemented with 150 mM NaCl, grown in the growth chamber under the same conditions as the Glc cotyledon greening assay, and analyzed for the percentage of surviving seedlings after 3 weeks. The experiments were conducted in triplicate for each line (50 seeds each).

### Measurement of Chlorophyll Content and Photochemical Efficiency (*F*_v_/*F*_m_)

Chlorophyll (Chl) content of leaves was determined by the spectrophotometric method (Lichtenthaler, 1987). Leaf powder was used to extract Chl pigments in 96% ethanol. Absorbance was analyzed at 648.6 and 664.2 nm. Chl fluorescence measurement was performed using a pulse-modulated fluorometer (Junior-PAM, Heinz Walz, Effeltrich, Germany). Photosystem (PS) II quantum yield (*F*_v_/*F*_m_) was calculated from the *F*_0_ and *F*_m_ values as described in the manufacturer’s instructions (Junior-PAM).

### Accession Numbers

Arabidopsis Genome Initiative numbers for the sequences used in this study are as follows: *bHLH34* (At3g23210), *AtPGR* (At5g19930), *bHLH104* (At4g14410), *bHLH105* (At5g54680), *bHLH115* (At1g51070), *bHLH28* (At5g46830), *bHLH47* (At3g47640), *bHLH11* (At4g36060), *bHLH121* (At3g19860), *AtHXK1* (At4g29130), *RAB18* (At5g66400), *RD29A* (At5g52310), *GIN6* (At2g40220), *AtAPR2* (At1g62180), *ABO3* (At1g66600), *ABI1* (At4g26080), *AtOZF2* (At4g29190), *RD29B* (At5g52300).

## Results

### Identification of the bHLH34 Candidate Related to *AtPGR* Expression

Recently, we demonstrated that the promoter sequences between nucleotide (nt) positions -999 and -456 of the *AtPGR* gene modulate transcription expression of the gene during Glc treatment (Chung et al., 2016). To identify transcription factors involved in the regulation of *AtPGR*, we performed yeast one-hybrid screening of a cDNA library of *Arabidopsis* (Chung et al., 2016). The yeast one-hybrid method involved in the *X*-galactosidase (Gal) filter assay was used to isolate transcription factor candidates that bind to P999-binding site (between positions -999 and -456 nt) in the upstream region of *AtPGR* (**Figure 1A** and Supplementary Figure S1A). Sequence analysis of the clone isolated from the P999 binding site (-999 to -456 nt) revealed that the gene encoded a basic helix-loop-helix (bHLH) family protein (At3g23210), which have been designated as a bHLH34 in *Arabidopsis* (Supplementary Figure S1B). *bHLH34* cDNA is 963 bp long and encodes a protein of 320 amino acid residues with a calculated molecular weight of 35.6 kDa, which shows 30–56% identity and 52–67% similarity with *Arabidopsis* proteins bHLH115, bHLH105, bHLH104, bHLH28, bHLH47, bHLH11, and bHLH121. A phylogenetic tree representing the distance groups of sequences was built using a cluster algorithm (Supplementary Figure S1C). bHLH34 harbors a conserved single DNA-binding domain within a basic helix-loop-helix motif in its central region that is 88, 76, and 69% identical to the corresponding region of *Arabidopsis* bHLH104, bHLH115, and bHLH105 proteins, respectively (Supplementary Figure S1D). Recently, Li et al. (2016) demonstrated that bHLH34 and bHLH104 modulate iron homeostasis in *Arabidopsis*. Protein interaction assays indicate that both heterodimers and homodimers can form among bHLH34, bHLH104, and bHLH105. The bHLH105, also known as IAA-LEUCINE RESISTANT 3 (ILR3), modulate metal homeostasis, which influences IAA-conjugate hydrolysis (Rampey et al., 2006).

### bHLH34 Interacts Specifically with the GAGA *Cis*-Element of *AtPGR* Promoter *in Vitro*

We attempted to express and purify the bHLH34 protein in *E. coli* to undertake electrophoretic mobility shift assay (EMSA) with the promoter in the region -999 to -456 of *AtPGR*. An EMSA was carried out to test whether the candidate protein bHLH34 can interact with each of the four DNA fragments (P1: -999 to -875 nt, P2: -874 to -735 nt, P3: -734 to -595 nt, and P4: -594 to -456; **Figure 1A**). Purified bHLH34 protein bound to all four fragments: P1, P2, P3, and P4 (**Figure 1B**).

To further investigate bHLH34-binding sites, we aligned the sequences of the P1-P4 fragments, and found that there were conserved *cis*-elements (5′-GAGA-3′) at six regions in the alignment (**Figure 1C**). The six DNA binding elements (DBEs), named as DBE1, DBE2, DBE3, DBE4, DBE5, or DBE6, were employed as a probe in an EMSA (**Figure 1C**). When we used the maltose-binding protein (MBP) as a negative control, there was no DNA–protein complex in the binding assay with DBE1 and DBE2 (**Figure 1D**, lane 3). Purified bHLH34 protein bound strongly to all six oligonucleotides DBE1–DBE6 (**Figure 1D**, lane 2). These results indicated that bHLH34 is a DNA-binding protein. To investigate that bHLH34 recognizes these oligonucleotides, excess unlabeled DBE1 was added. An addition of a molar excess of the unlabeled DBE1 oligonucleotide probe gradually diminished the shifted bands of bHLH34 protein in a dose-dependent manner (**Figure 1E**). These findings indicate that bHLH34 recognizes the six DBE fragments containing the consensus GAGA sequence.

To verify the specificity of binding between bHLH34 and the GAGA element, an EMSA was performed using various mutated DBE1 oligonucleotide fragments (**Figure 1F**). Mutations introduced in the GAGA sequence abrogated the binding of bHLH34 (**Figure 1F**, lanes m1, m2, m3, m4, and m5). From these results, we concluded that bHLH34 has sequence-specific binding affinity for the GAGA element *in vitro*.

On the other hand, consensus DNA sequence recognized by the bHLH proteins is known well as the E-box (5′-CANNTG-3′) (Robinson et al., 2000) and, single copy E-box (between positions -788 and -783 nt) exists in P999 site of the *AtPGR* gene. To determine whether bHLH34 protein could recognize the E-box, we conducted the EMSA assay with probe containing the E-box sequence (5′-CACTTG–3′; Supplementary Table S1), showing that bHLH34 could bind to E-box sequence (**Figure 1G**). This result indicate that bHLH34 binds to not only GAGA *cis*-element but also E-box sequence.

### bHLH34 Activates the Expression of the *AtPGR*

**FIGURE 2:**
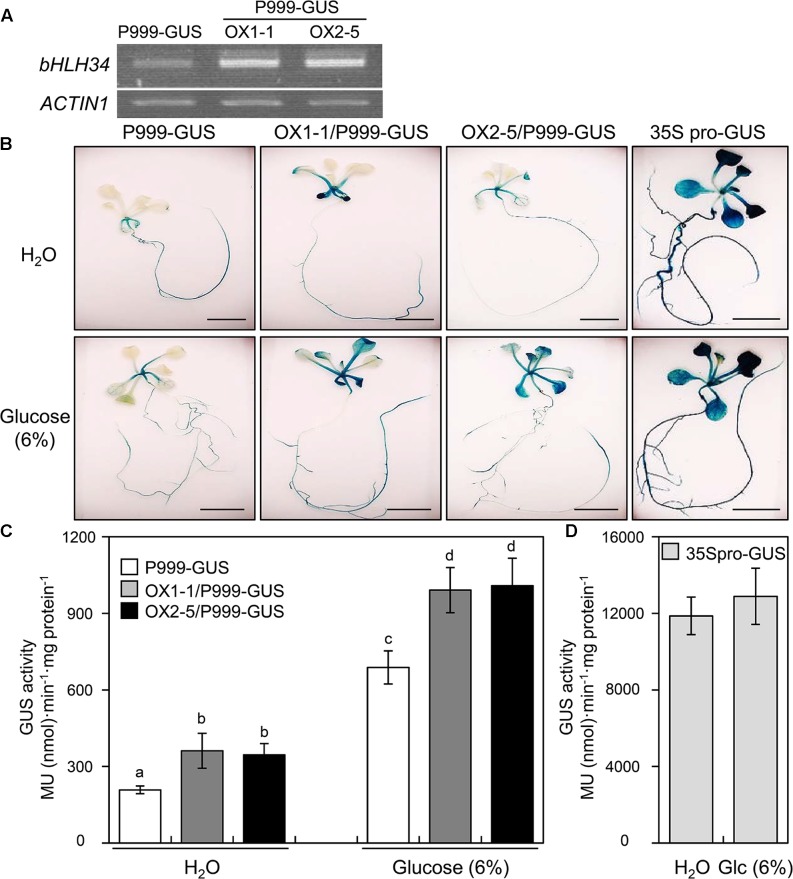
bHLH34 activates *AtPGR* expressions. **(A)** Expression levels of *bHLH34* in P999-GUS and two independent P999-GUS/*bHLH34*-overexpressing (OX1-1/P999-GUS, OX2-5/P999-GUS) transgenic plants were confirmed by reverse transcription (RT)-PCR using RNA extracted from 14-day-old seedlings. *Actin 1* served as an internal RT-PCR control. **(B)** The activities of the 999-bp regulatory promoter region (P999) of the *AtPGR* gene were examined by means of GUS as a reporter in transgenic plants (P999-GUS). These transgenic plants overexpressing *bHLH34* (OX1-1/P999-GUS, OX2-5/P999-GUS) were analyzed histochemically by treatment with H_2_O or 6% glucose for 12 h. 35S pro-GUS served as a positive control for analysis of GUS activity. Scale bars = 5 mm. **(C,D)** P999-GUS, OX1-1/P999-GUS, OX2-5/P999-GUS **(C)**, and 35S pro-GUS **(D)** seedlings grown on the MS medium for 12 days were carefully taken out and treated with H_2_O or 6% Glc for 12 h. Subsequently, seedlings were subjected to GUS staining, and GUS activity was measured. The values of GUS activities are averages of three independent enzymatic assays. Each assay was performed with extracts obtained from three individual seedlings of each transgenic plant. Error bars indicate standard deviations, and different letters above bars indicate a statistical difference (ANOVA, *P* < 0.05).

To determine the transcriptional activity of *AtPGR* by the bHLH34, we examined the *AtPGR* expression level in P999-GUS transgenic plants, when the *bHLH34* was constitutively expressed under the control of Cauliflower Mosaic Virus (CaMV) 35S promoter (OX1-1/P999-GUS, OX2-5/P999-GUS). Two independent lines showing high levels of *bHLH34* transgene expression (**Figure 2A**) were selected for transcription activity analyses. As shown in **Figures 2B,C**, under normal conditions, OX1-1/P999-GUS and OX2-5/P999-GUS lines increased GUS expression to approximately double that of P999-GUS alone (1.73- and 1.66-fold, respectively). When 6% Glc was applied to seedlings for 12 h, GUS activity in the P999-GUS transgenic plant was elevated in the leaves and roots (**Figure 2B**). In addition, a significant increase in GUS activity levels (1.44- to 1.47-fold) was observed in P999-GUS transgenic lines overexpressing *bHLH34* constructs in comparison with the P999-GUS transgenic plant (**Figures 2B,C**). Furthermore, to investigate the level and tissue specificities of the *AtPGR* expression, we measured GUS activity and the transcript level of *AtPGR* on root and leaf tissues of the P999-GUS transgenic plants treated with or without Glc. As shown in Supplementary Figure S2, GUS and *AtPGR* expressions analyses indicated that the P999-GUS transgenic lines were expressed more strongly in leaves than in roots under the same condition. Constitutive CaMV35S promoter (35S pro)-GUS served as a positive control for analysis of GUS activity (**Figures 2B,D**). Taken together, these data indicate that bHLH34 is a functional transcription factor that regulates the expression of the *AtPGR* gene. Moreover, it appears that bHLH34 functions as a transcriptional activator.

### *bHLH34* Expression in Various Organs and Different Developmental Stages

**FIGURE 3:**
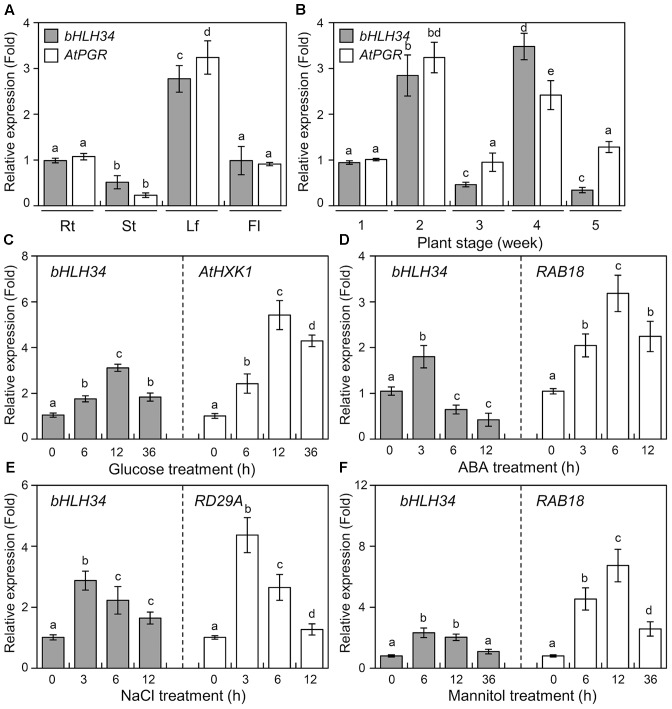
Expression of *bHLH34* in *Arabidopsis*. **(A,B)** Expression profiles for *AtPGR* gene in various organs and at different developmental stages. Results are relative to the *bHLH34* expression and represent the average of three independent biological replicates (*n* = 5–25, mean values ± SD, ANOVA, *P* < 0.05). Different letters above bars indicate a statistical difference. **(A)** RNA levels were confirmed by qPCR using total RNA isolated from roots (Rt), stems (St), leaf (Lf), and flowers (Fl). **(B)** RNA levels were determined by qPCR using total RNA isolated at the indicated plant sampling stages. **(C–F)** Expression of the *bHLH34* in *Arabidopsis* under glucose, ABA, NaCl, and mannitol stress conditions. qPCR analyses of the expression of *bHLH34* involved in glucose **(C)**, ABA **(D)**, NaCl **(E)**, or mannitol **(F)** responses. Total RNA samples were obtained from 14-day-old seedlings treated with 6% glucose, 100 μM ABA, 150 mM NaCl, or 400 mM mannitol at the indicated time points. Error bars indicate standard deviations of three independent experiments, and different letters above bars indicate a statistical difference (ANOVA, *P* < 0.05). Each experiment was performed with total RNA of each sample obtained from 20 seedlings. *Arabidopsis Actin 1* was used as the internal control. *HXK1* **(C)**, *RAB18* **(D)**, *RD29A* **(E)**, or *RAB18* **(F)** gene served as a control for the glucose, ABA, salt, or mannitol stress treatment, respectively.

To examine the tissue expression pattern of the *bHLH34* and *AtPGR*, we analyzed the expression patterns of the *bHLH34* and *AtPGR* in various organs and at different developmental stages of *Arabidopsis* seedlings by quantitative real-time PCR (qPCR). The maximal expression levels of the *bHLH34* and *AtPGR* were detected in leaves, while moderate expression levels were detected in roots and flowers; low expression levels were detected in stem (**Figure 3A**). The expression patterns of *bHLH34* and *AtPGR* during leaf development from 1 week after germination (WAG) to 5 WAG are also examined (**Figure 3B**). The expression patterns of *bHLH34* and *AtPGR* were similar at the 1 and 2 WAG stages. In contrast, different expression patterns were observed for *bHLH34* and *AtPGR* at the 3, 4 and 5 WAG stages, indicating that the expression level of the *AtPGR* is likely to be involved other unidentified regulatory factors or be independent of *bHLH34* at different developmental stage.

### The *bHLH34* Gene Is Regulated by Abiotic Stress

To determine the *in vivo* functions of bHLH34, the accumulation of *bHLH34* mRNA was assessed in *Arabidopsis* using qPCR during Glc, ABA, salt, and mannitol treatment. *bHLH34* was induced in *Arabidopsis* seedlings after Glc treatment, and the *bHLH34* transcript reached its maximum amount at 12 h followed by a slight decline (**Figure 3C**). In contrast, only slight upregulation of *bHLH34* was observed after 3 h of ABA treatment (**Figure 3D**). In particular, transcript level of *bHLH34* reached a peak within 3 and 6 h after the salt and mannitol treatment, respectively, and decreased for the remainder of the salt and mannitol experiments (**Figures 3E,F**). The stress-inducible *A. thaliana Hexokinase 1* (*AtHXK1*), *Responsive to ABA 18* (*RAB18*), and *Responsive to Desiccation 29A* (*RD29A*) genes served as references for the abiotic stress treatment (**Figures 3C–F**). These results indicate that *bHLH34* is modulated by Glc, ABA, salinity, and osmotic stress.

### Abiotic Stress Responses in *bHLH34* Transgenic Plants

To investigate *bHLH34* function *in vivo*, *bHLH34* was overexpressed in *Arabidopsis* transgenic plants under the control of the 35S promoter. Twelve homozygous lines (T_3_ generation) were obtained, and two independent lines (OX2-1 and OX3-5) showing high levels of transgene expression (Supplementary Figure S3A) were selected for phenotypic characterization. To further evaluate the functional consequences of the loss of *bHLH34*, *bhlh34* RNA interference (RNAi) lines were generated using the exon 1 to exon 2 cDNA sequence. *bHLH34* expression was assessed by qPCR in two independent *bhlh34* RNAi (*ri2-2* and *ri5-1*) lines. *bHLH34* expression was found to be knocked down in the RNAi lines (Supplementary Figure S3A). In addition, to determine whether the *bHLH34* RNAi construct influences on the expression levels of the *bHLH34* homologs, *bHLH104*, *bHLH105* and *bHLH115*, in *bhlh34* RNAi lines, we performed qPCR assay. The assay revealed that expression of the *bhlh34* RNAi construct do not inhibit the expression *bHLH104*, *bHLH105* and *bHLH115*, indicating that *bhlh34* RNAi construct is highly specific to its own target transcripts (Supplementary Figures S3B–D). No morphological differences were observed among WT, *bHLH34*-overexpressing and *bhlh34* RNAi plants, when grown on the Murashige and Skoog (MS) medium containing 1% sucrose (Murashige and Skoog, 1962) (Supplementary Figures S3E–G).

**FIGURE 4:**
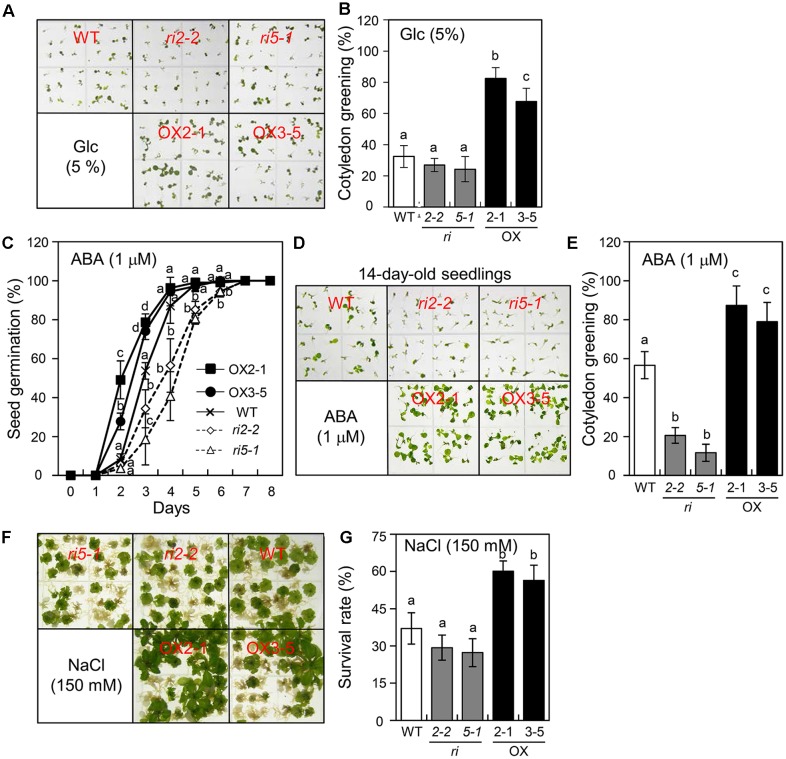
The influence of *bHLH34* transgenic plants on glucose (Glc), ABA, and salt stress insensitivity. **(A,B)** Effects of Glc treatment on cotyledon greening. Seeds of the samples were sown on MS agar plates supplement with 5% Glc and allowed to grow for 10 days. The photograph shows that *bHLH34*-overexpressing lines (OX2-1 and OX3-5) were better development and greener than the WT and *bhlh34* RNAi (*ri2-2* and *ri5-1*) plants under Glc stress condition **(A)**. Seedlings with green cotyledons were counted (triplicates, *n* = 50 each). Error bars indicate standard deviations, and different letters above bars indicate a statistical difference (ANOVA, *P* < 0.05) **(B)**. **(C)** Sensitivity of germination to ABA. Seeds were sown on MS agar plates supplement with 1 μM ABA and allowed to grow for indicated days, and germination was counted (triplicates, *n* = 50 each). Error bars indicate standard deviations for three independent experiments, and different letters above bars indicate a statistical difference (ANOVA, *P* < 0.05). **(D)** Seeds of the samples were sown on the MS medium containing 1 μM ABA and permitted to grow for 14 days. The photograph shows that *bHLH34*-overexpressing lines were greener than the WT and *bhlh34* RNAi plants under ABA condition. **(E)** Sensitivity of cotyledon greening to ABA. Seeds were sown on the MS medium containing 1 μM ABA and permitted to grow for 14 days, and seedlings with green cotyledons were counted (triplicates, *n* = 50 each). Error bars indicate standard deviations for three independent experiments, and different letters above bars indicate a statistical difference (ANOVA, *P* < 0.05). **(F)** Effects of salt stress on plant growth. Seeds were sown on MS medium containing 150 mM NaCl and permitted to grow for 21 days. The photograph shows that *bHLH34*-overexpressing lines were better development than the WT and *bhlh34* RNAi plants under salt stress condition. **(G)** Effects of salt treatment on cotyledon greening. Seeds were sown on MS medium supplement with 150 mM NaCl and allowed to grow for 21 days, and surviving plants was counted (triplicates, *n* = 35 each). Error bars indicate standard deviations, and different letters above bars indicate a statistical difference (ANOVA, *P* < 0.05).

Chung et al. (2011) demonstrated that AtPGR is required for the modulation of *Arabidopsis* insensitivity to Glc exposure. To determine whether *bHLH34* is also related to the Glc response, Glc sensitivity was evaluated by measuring the rate of cotyledon greening. WT, *bHLH34*-overexpressing (OX2-1, OX3-5), and *bhlh34* (*ri2-2*, *ri5-1*) seeds were grown on the MS medium containing 5% Glc for 10 days. The cotyledon greening rate of WT seedlings was 33%, and this rate was approximately 25.6% (range 24.3–26.9%) for *bhlh34* RNAi lines (**Figures 4A,B**). In contrast, approximately 75% (range 67.6–82.4%) of the *bHLH34*-overexpressing cotyledons expanded and turned green (**Figures 4A,B**). To assess the effects of *bHLH34* expression on the ABA response, the seeds of the WT, *bhlh34*, and *bHLH34*-overexpressing plants were germinated on the MS medium containing 1% sucrose and 1 μM ABA. The germination percentage of *bhlh34* lines was much more affected than that of WT and *bHLH34*-overexpressing plants by treatment with 1 μM ABA. The germination rate of *bHLH34*-overexpressing plants was higher than that of the WT (**Figure 4C**). These results show that the bHLH34 is required for ABA-modulated seed germination in *Arabidopsis*.

The ABA-induced effect was also evaluated by measuring the cotyledon greening rate. The relative reduction in cotyledon greening of the *bhlh34* lines in response to ABA was more profound than that of the WT and *bHLH34*-overexpressing plants at 14 days after germination. In the WT, the cotyledon greening efficiency was 56.7%, whereas the cotyledon greening efficiency of *bHLH34*-overexpressing plants was 78.9–87.4% during treatment with ABA. In contrast, the cotyledon greening efficiency of *bhlh34* RNAi lines was 11.7–20.6% (**Figures 4D,E**). These results indicated that the *bhlh34* RNAi lines were more likely to be sensitive to Glc and ABA than the WT plant was. On the other hand, the *bHLH34*-overexpressing plants were less sensitive to exogenous Glc and ABA than were the WT and *bhlh34* RNAi plants.

To characterize the effects of salt stress on the *bHLH34*-overexpression plants, we evaluated the response to treatment with 150 mM NaCl. The survival rate of the WT was slightly above 35% at 3 weeks after germination. Fewer than 30% of plants of the *bhlh34* RNAi line (*ri2-2*) remained alive, and 27% of the *bhlh34* leaves (*ri5-1*) turned green, as compared with 56–60% in OX2-1 and OX3-5 (**Figures 4F,G**). These results are consistent with the idea that bHLH34 is necessary for the regulation of developmental growth under abiotic stress.

### *AtPGR* Expression in *bHLH34* Transgenic Plants upon Glc Treatment

**FIGURE 5:**
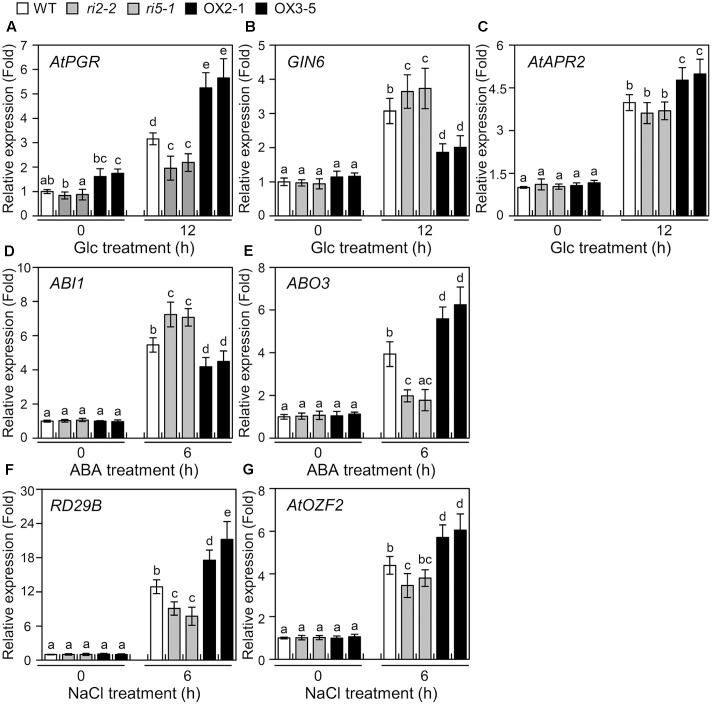
Expression of abiotic stress-regulated genes in *bHLH34* transgenic plants. **(A–G)** mRNA levels of *AtPGR* **(A)**, *GIN6* **(B)**, *AtAPR2* **(C)**, *ABI1* **(D)**, *ABO3* **(E)**, *RD29B* **(F)**, or *AtOZF2* **(G)** were measured by qPCR using total RNA from 14-day-old WT, two independent *bhlh34* RNAi (*ri2-2*, *ri5-1*), and two independent *bHLH34*-overexpressing (OX2-1, OX3-5) seedlings, which were treated with 6% Glc, 100 μM ABA, or 150 mM NaCl with gentle shaking for the indicated period. The mean value of three technical replicates was normalized to the level of *Actin 1* mRNA, an internal control. Error bars indicate standard deviations, and different letters above bars indicate a statistical difference (*n* = 20 each, ANOVA, *P* < 0.05).

As shown in **Figures 2B,C**, the induction of GUS activity caused by bHLH34 upon exposure to exogenous Glc may indicate that AtPGR participates in the Glc response through a bHLH34-mediated signaling pathway. To quantify *AtPGR* expression in the *bHLH34* transgenic plants during Glc treatment, a second set of experiments using qPCR was conducted. **Figure 5A** shows that *AtPGR* mRNA expression in *bHLH34*-overexpressing transgenic plants without Glc treatment appears to be higher than that in WT and *bhlh34* RNAi lines. By contrast, the transcript abundance of Glc-inducible *AtPGR* showed greater reduction in *bhlh34* RNAi (*ri2-2* and *ri5-1*) plants as compared with WT after Glc treatment. As expected, *AtPGR* expression was more strongly induced by Glc treatment in the *bHLH34*-overexpressing transgenic (OX2-1 and OX3-5) lines than in the WT and *bhlh34* RNAi plants. Basically, these results are consistent with those obtained in the GUS activity assay after Glc treatment (**Figure 2**).

### Expression of Stress-Regulated Genes in *bHLH34* Transgenic Plants

To investigate the transcriptional regulatory roles of bHLH34, we screened genes involved in Glc, ABA, and salt stress response and harboring the promoter including GAGA *cis*-element and E-box sequences. Evaluated were six genes, *Glucose-Insensitive 6* (*GIN6*), *Arabidopsis thaliana Adenosine 5′-Phosphosulfate Reductase 2* (*AtAPR2*), *Abscisic acid Insensitive 1* (*ABI1*), *ABA Overly Sensitive 3* (*ABO3*), *Responsive to Desiccation 29B* (*RD29B*), and *A. thaliana Oxidation-related Zinc Finger 2* (*AtOZF2*), which are induced by various abiotic stresses (Leung et al., 1997; Arenas-Huertero et al., 2000; Ren et al., 2010; Huang et al., 2012; Chung et al., 2015).

As shown in **Figures 5B–F**, Glc- or ABA-induced expression of *GIN6* or *ABI1* was significantly reduced in *bHLH34*-overexpressing lines (OX2-1, OX3-5), in comparison with WT and *bhlh34* RNAi (*ri2-2*, *ri5-1*) plants (**Figures 5B,D**). However, the induction of transcript levels of *AtAPR2* and *ABO3* were more pronounced in *bHLH34*-overexpressing lines than in WT and *bhlh34* RNAi plants during Glc or ABA conditions (**Figures 5C,E**). The induction of transcript levels of dehydration-inducible *RD29B* and *AtOZF2* genes were more pronounced in *bHLH34*-overexpressing lines than in WT and *bhlh34* RNAi lines, but less pronounced in the *bhlh34* RNAi lines than in WT following salt treatment (**Figures 5F,G**). These results indicated that bHLH34 may act positively or negatively depending on the types of abiotic stress-related genes harboring the promoter including GAGA *cis*-element and E-box sequences.

### Genetic Interaction between *bHLH34* and *bHLH104* in Glc Response

**FIGURE 6:**
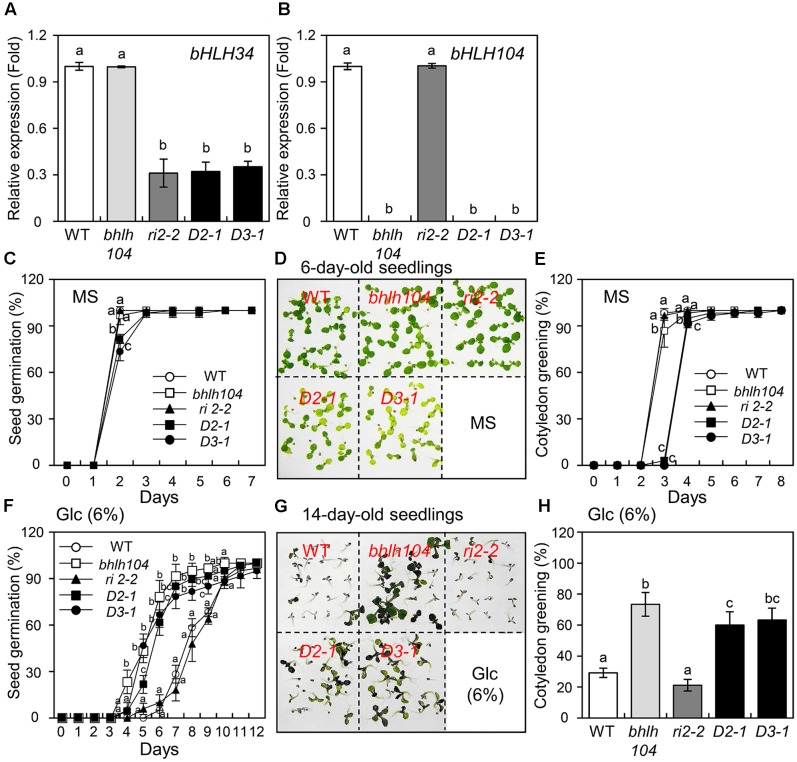
Genetic analysis of *bhlh104*, *bhlh34* RNAi (*ri2-2*) and *bhlh34*/*bhlh104* double mutants (*D2-1* and *D3-1*) under normal and glucose (Glc) conditions. **(A,B)** *bHLH34* **(A)** and *bHLH104* **(B)** expression levels in WT, *bhlh104*, *bhlh34*, and two independent *bhlh34*/*bhlh104* mutant lines (*D2-1* and *D3-1*) were confirmed by qPCR using RNA extracted from 10-day-old seedlings. The mean value of three technical replicates was normalized to the level of *Actin 1* mRNA, an internal control. Error bars indicate standard deviations, and different letters above bars indicate a statistical difference (*n* = 20 each, ANOVA, *P* < 0.05). **(C)** Seed germination assays in the normal condition. Seeds of WT, *bhlh104*, *bhlh34* RNAi (*ri2-2*) and *bhlh34*/*bhlh104* mutants (*D2-1* and *D3-1*) were sown on the sterile MS medium and permitted to grow for indicated days, germination was scored (triplicates, *n* = 50 each). Error bars indicate standard deviations for three independent experiments, and different letters above bars indicate a statistical difference (50 seeds per point, ANOVA, *P* < 0.05). **(D)** Seeds of the samples were sown on the MS medium and permitted to grow for 6 days. The photograph indicates that *bhlh104* showed development and green cotyledons similar to those of WT and *bhlh34*, while *bhlh34*/*bhlh104* lines had paler green leaves. **(E)** Cotyledon greening assays in the normal condition. Seeds were sown on the MS medium and permitted to grow for indicated days, green cotyledons were counted (triplicates, *n* = 50 each). Error bars indicate standard deviations for three independent experiments, and different letters above bars indicate a statistical difference (50 seeds per point, ANOVA, *P* < 0.05). **(F)** Effects of Glc treatment on seed germination. Seeds were sown on the MS medium containing 6% Glc and permitted to grow for indicated days, germination was scored (triplicates, *n* = 50 each). Error bars indicate standard deviations for three independent experiments, and different letters above bars indicate a statistical difference (50 seeds per point, ANOVA, *P* < 0.05). **(G)** Seeds of the samples were sown on the MS medium and permitted to grow for 14 days. The photograph shows that *bhlh104* and *bhlh34*/*bhlh104* mutant lines show better development and are greener than the WT and *bhlh34* at high Glc concentration. **(H)** Effects of Glc treatment on cotyledon greening. Seeds were sown on the MS medium and permitted to grow for 14 days, green cotyledons were counted (triplicates, *n* = 50 each). Error bars indicate standard deviations for three independent experiments, and different letters above bars indicate a statistical difference (ANOVA *P* < 0.05).

bHLH34 and bHLH104 sequences are highly homologous, with a significant 56% identity at the amino acid level (Supplementary Figure S4). Furthermore, bHLH34 and bHLH104 harbor a conserved DNA-binding domain within the basic helix-loop-helix motif in their central region that is 88% identical between these proteins (Supplementary Figure S1D). To determine whether the *bHLH104* (*At4g14410*) gene is related to Glc, we obtained the *At4g14410*-tagged T-DNA insertion mutant SALK_005802. The absence of *bHLH104* transcript was verified using qPCR (**Figure 6B**). The mutant was designated as *bhlh104*.

To investigate the genetic relation between *bHLH34* and *bHLH104*, a *bHLH34* RNAi construct was transformed into the *bhlh104* mutant. Ten homozygous lines (T_3_ generation) were obtained, and *bHLH34* expression was assessed by qPCR in two independent *bhlh34*/*bhlh104* lines (*D2-1* and *D3-1*). *bHLH34* expression was knocked down successfully in the double mutant lines (**Figure 6A**), and the effects of Glc on seed germination or cotyledon greening efficiency in the WT, *bhlh34* RNAi (*ri2-2*), *bhlh104*, and *bhlh34*/*bhlh104* (*D2-1* and *D3-1*) lines were assessed. Under normal condition, the germination percentage and cotyledon greening rate were similar between WT, *bhlh34*, and *bhlh104* mutant on the MS medium (**Figures 6C–E**). In contrast, the germination percentage or cotyledon greening rate of *bhlh34*/*bhlh104* lines was much more affected than that of WT, *bhlh34*, and *bhlh104* at 2 days after germination (**Figure 6C**). In addition, 6-day-old *bhlh34*/*bhlh104* lines displayed paler green leaves (**Figures 6D,E**), more strongly decreased chlorophyll content, and lower photosynthetic efficiency (*F*_v_/*F*_m_) in comparison with the WT, *bhlh34*, and *bhlh104* mutant, implying that the *bhlh34*/*bhlh104* double mutant had a chlorotic leaf phenotype (Supplementary Figure S5). These results suggest that both bHLH34 and bHLH104 are most likely involved in seed germination and leaf chlorophyll synthesis in *Arabidopsis*.

To characterize the correlation of bHLH34 and bHLH104 functions in the Glc response, seeds of WT, *bhlh34* RNAi (*ri2-2*),*bhlh104*, and *bhlh34*/*bhlh104* lines (*D2-1* and *D3-1*) were germinated on the MS medium containing Glc. At 6% Glc, 28.3% of WT and 18.1% of *bhlh34* seeds germinated at 7 days, and this rate increased to 91.7% and approximately 81.7% (range 78.3–85%) for *bhlh104* and *bhlh34*/*bhlh104* seeds, respectively (**Figure 6F**). When the *bhlh34*/*bhlh104* lines were allowed to grow for 14 days prior to the assessment of cotyledon greening rates in response to Glc, 23.3% of WT and 21.2% of *bhlh34* leaves expanded and turned green, as compared to 73.3% in the *bhlh104* mutant. Furthermore, approximately 62.7% (range 60–63.3%) of two *bhlh34*/*bhlh104* lines survived at 14 days (**Figures 6G,H**). These results show that *bhlh104* and *bhlh34*/*bhlh104* mutants are more likely to be insensitive to Glc than the WT and *bhlh34* RNAi. This finding indicated that the *bhlh104* mutant is epistatic to the *bhlh34* under high Glc condition, and bHLH104 may modulate the sensitivity of seed germination and cotyledon greening phenotype more tightly than bHLH34 does in the Glc response as a negative regulator.

## Discussion

Glucose signaling has been implicated in regulation of plant growth and development. To date, no common or conserved *cis*-element to regulate the Glc-responsive genes has been reported (Rolland et al., 2006). These findings are key to the identification of specific transcription factors in Glc signaling. In addition, Glc signaling is associated with ABA biosynthesis and signaling (Finkelstein and Gibson, 2001; Rook et al., 2006), and other processes affected by ABA, such as osmotic stress (Rolland et al., 2002).

In this study, a transcription factor involved in the Glc response, bHLH34, was isolated by yeast one-hybrid screening (Supplementary Figure S1A) and was found to be capable of modulating the Glc response through the regulation of *AtPGR* expression. As shown in **Figure 2**, *bHLH34*-overexpressing constructs in P999-GUS transgenic lines increased GUS activity more strongly during Glc treatment than those without Glc exposure. In addition, the level of GUS activities in leaves of both P999-GUS and P999-GUS/*bHLH34*-overexpressing plants was stronger than those in roots regardless of Glc treatment (Supplementary Figure S2A). The expression levels of Glc-inducible *AtPGR* showed enhanced induction in *bHLH34*-overexpressing transgenic plants relative to the WT and *bhlh34* RNAi plants during Glc treatment. Nevertheless, *AtPGR* expression was decreased more strongly by Glc treatment in *bhlh34* RNAi transgenic lines than in WT plants (**Figure 5A**). These results suggest that bHLH34 acts as an activator of the *AtPGR* gene in Glc signaling. EMSA results showed that bHLH34 binds to the GAGA and E-box sequences in the upstream promoter region of the *AtPGR* gene (**Figure 1**). Previously, it was reported that AtMYC2 (bHLH family protein) can bind to both G-box (CACGTG) and Abscisic acid-responsive element (ABRE, ACGTGG/TC) (Abe et al., 2003). Thus, a coupling *cis*-element is required to regulate some ABA-responsive genes. Therefore, it can be implied that bHLH34 binds to the GAGA *cis*-element and E-box sequence as a coupling element to regulate the *AtPGR* gene.

Several sugar-responsive promoters were shown to contain the carbohydrate metabolite signal-responsive element (CMSRE)-1 (TGGACGGCC), TATCCA box, translation elongation factor (TEF) element (CATAAT), and SUC-6 element (GAANGAGANGA) (Lu et al., 2002; Morikami et al., 2005; Geisler et al., 2006; Li et al., 2006). No conserved *cis*-acting element has been reported, indicating perhaps the complex nature of sugar signaling pathways. Moreover, the SUC-6 box has a sequence similar to that of the GAGA box (bHLH34-binding site), indicating that the SUC-6 *cis*-element may be required for the activation of gene expression through competition for the binding site with bHLH34 or other bHLH domain transcription factors. Although the gene-regulatory mechanism is still unknown, we propose that bHLH34 may affect downstream genes involved in sugar signaling.

At present, it is known that plant bHLHs are involved in developmental processes such as development of an embryo, root hair, stomata, carpel margin, petals, and anther (Pires and Dolan, 2010). Furthermore, numerous bHLHs play roles in the regulation of light (Castelain et al., 2012) and hormonal signaling, such as ABA, brassinosteroid, jasmonate, ethylene, and gibberellin signaling pathways (Friedrichsen et al., 2002; Abe et al., 2003; Song et al., 2014). Recent research revealed that overexpression of some *bHLH* genes elevates tolerance to freezing, cold, pathogen, salinity, oxidative and osmotic stresses (Chinnusamy et al., 2003; Babitha et al., 2013; Fan et al., 2014; Liu et al., 2015), indicating that bHLHs are closely associated with the responses to biotic and abiotic stress. Here, we demonstrated that *bHLH34*-overexpressing transgenic lines were more resistant to Glc, ABA, and salinity stress than were the WT and *bhlh34* RNAi plants, whereas *bhlh34* RNAi lines showed increased sensitivity to Glc, ABA, and salinity stress during cotyledon greening (**Figure 4**). These data imply that bHLH34 is a positive regulator of the Glc and abiotic stress responses.

It is widely known that Glc signaling can link ABA and a salinity response (Quesada et al., 2000; Rolland et al., 2002). The transcript of *bHLH34* is regulated not only by Glc but also by ABA, salt and osmotic stress (**Figure 3**), implying that bHLH34 possibly regulates a plant abiotic stress response in a Glc-dependent manner. To investigate this possibility, the expression of several stress-related genes (*GIN6*, *AtAPR2*, *ABI1*, *ABO3*, *AtOZF2*, and *RD29B*) was monitored in WT and *bHLH34* transgenic plants with and without Glc, ABA, or NaCl treatment. **Figure 5** showed that bHLH34 positively regulates *AtAPR2*, *ABO3*, *AtOZF2*, and *RD29B* but negatively regulates genes *GIN6* and *ABI1*. This result may be the reason for the increased resistance to abiotic stresses in *bHLH34*-overexpressing transgenic plants. Therefore, it is possible that bHLH34 is an important regulator in Glc dependent-mediated stress signaling responses or that it indirectly exerts the effect on the ABA and salt stress responses.

Considering the high similarity between bHLH34 and bHLH104 (Supplementary Figure S4), it was expected bHLH34 and bHLH104 to perform a similar function in the Glc response. In the present study, the comparison of phenotypes of *bhlh34*/*bhlh104* double mutants with WT, *bhlh34*, and *bhlh104* mutant revealed delayed seed germination, delayed cotyledon greening, paler green leaves, decreased total chlorophyll contents, and reduced photosynthetic efficiency when grown under normal condition (**Figure 6** and Supplementary Figure S5), suggesting that the bHLH34-bHLH104 complex may be involved in photosynthesis and development processes. As shown in **Figure 6**, the germination or cotyledon greening percentage of *bhlh104* and *bhlh34*/*bhlh104* double mutant lines was higher than that of the WT and *bhlh34* RNAi upon the treatment with high Glc concentration, indicating that *bhlh34*/*bhlh104* double mutant phenocopied Glc insensitivity of the *bhlh104* mutant. Thus, bHLH104 is epistatic to bHLH34. Therefore, it is possible that bHLH104 regulates the Glc response more tightly than bHLH34 does. Recently, Li et al. (2016) reported that bHLH34 and bHLH104 positively regulate iron homeostasis in *Arabidopsis* as a homo- and heterodimer. However, here, bHLH34 and bHLH104 displayed different function in Glc signaling, implying that bHLH34 and bHLH104 can be possible to bind to different *cis*-element for Glc signaling. Therefore, we need to further study *cis*-element of bHLH104 for Glc signaling. Overall, our results suggest that the bHLH34-bHLH104 complex plays a significant role in modulating Glc-dependent early seedling development and the Glc signaling.

## Author Contributions

J-HM and CK designed the experiments and interpreted the results. SL provided technical assistance with the EMSA analysis. J-HM, H-WJ, DY, and K-HL carried out the experiments and interpreted the results. CK supervised the project and complemented the writing.

## Conflict of Interest Statement

The authors declare that the research was conducted in the absence of any commercial or financial relationships that could be construed as a potential conflict of interest.
