# Supplementary material for: Arabidopsis Basic Helix-Loop-Helix 34 (bHLH34) Is Involved in Glucose Signaling through Binding to a GAGA Cis-Element
Source: Front Plant Sci. 2017 Dec 11;8:2100. doi: 10.3389/fpls.2017.02100 (PMC5732184; doi:10.3389/fpls.2017.02100)
Supplement: Supplementary file 1 [file Presentation_1.PDF]

Supplementary Materials

Arabidopsis basic Helix-Loop-Helix 34 (bHLH34) is involved in glucose signaling through binding to a GAGA *cis*-element

Ji-Hee Min[1], Hyun-Woo Ju[1], Dayoung Yoon[1], Kyeong-Hwan Lee[2], Sungbeom Lee[3], and Cheol Soo Kim[1,*]

***Correspondence:** Cheol Soo Kim : cskim626@jnu.ac.kr

Supplementary Materials and Methods

Overexpression of *bHLH34* in P999-GUS transgenic plants and analysis of GUS activity

Generation of the *AtPGR* promoter 999 (P999)-GUS construct was conducted as described before (Chung et al., 2016). To construct the overexpression of *bHLH34* in P999-GUS transgenic lines, the full-length *bHLH34* cDNA (*At3g23210*) was amplified using RT-PCR, and the generated product was cloned into the pDONR/ZEO vector for DNA sequence analysis. The RT-PCR primers were as follows: forward 5′-GGGGACAAGTTTGTACAAAAAAGCAGGCTTCATGTATCCATCAATCGAAGACGA-3′ and reverse 5′-GGGGACCACTTTGTACAAGAAAGCTGGGTCAGCAACAGGAGGAAGATTTTTGA-3′. Amplification proceeded for 30 cycles consisting of 94 °C for 30 s; 57 °C for 30 s; and 72 °C for 1 min. The DNA fragment was then cloned into the plant expression vector pEarlyGate202 (Earley et al., 2006) by the Gateway system according to the manufacturer’s instruction (Invitrogen). After that, the resultant construct was introduced into the

Agrobacterium tumefaciens strain GV3101. These transformants were then introduced into the P999-GUS transgenic plants to generate the *bHLH34*-overexpressing/P999-GUS lines via *in planta* vacuum infiltration (Bechtold and Pelletier, 1998). T_3 homozygous transgenic lines (OX1-1/P999-GUS, OX2-5/P999-GUS) were selected for GUS analysis. Phosphinothricin (Duchefa, Haarlem, Netherlands) resistance of the T_2 generation from these selected lines segregated as a single locus. Constitutive CaMV35S promoter (35S pro)-GUS served as a positive control for analysis of GUS activity. We obtained 35S pro-GUS transgenic seeds from Dr. J.I. Kim (Han et al., 2015).

Histochemical staining in transgenic plants for GUS activity was conducted as described elsewhere (Jefferson et al., 1987). Briefly, whole seedlings were immersed in a 1 mM 5-bromo-4-chloro-3-indolyl-β-glucuronic acid (X-Gluc) solution in a buffer consisting of 100 mM sodium phosphate, pH 7.0, 0.5 mM potassium ferrocyanide, 0.5 mM potassium ferricyanide, 10 mM EDTA, and 0.1% Triton X-100, and then incubated for 4 h at 37 °C. Chlorophyll was removed from the plant tissues by immersion in 70% ethanol. To measure the strength of the GUS activity in the OX1-1/P999-GUS and OX2-5/P999-GUS transgenic lines, a fluorimetric assay was carried out with 4-methylumbelliferyl β-D-glucuronide (Bio Basic Inc., Markham, Ontario, Canada) as a substrate.

Generation of *bHLH34* transgenic lines

Total RNA samples were isolated from *Arabidopsis* leaves using the TRIzol reagent (Invitrogen, Carlsbad, CA, USA). RT-PCR was used to obtain full-length *bHLH34* cDNA. The RT-PCR primers were as follows: for *bHLH34*, forward primer 5′-GGGGACAAGTTTGTACAAAAAAGCAGGCTTCATGTATCCATCAATCGAAGACGA-3′ and reverse primer 5′-

GGGGACCACTTTGTACAAGAAAGCTGGGTCAGCAACAGGAGGAAGATTTTTGA-3′. Amplification proceeded for 35 cycles, with each cycle at 94 °C for 30 s, 57 °C for 30 s, and 72 °C for 1 min. The amplicon was then cloned into the pDONR/ZEO vector. Nucleotide sequences of new constructs were confirmed by DNA sequencing. The DNA fragment was then cloned into the plant expression vector pGWB514 vector (Nakagawa et al., 2007) by the Gateway system according to the manufacturer’s instruction (Invitrogen). After that, the resultant construct was introduced into the *A. tumefaciens* strain GV3101 via *in planta* vacuum infiltration. Homozygous lines (T_3 generation) from 12 independent transformants were obtained, and two lines for the *bHLH34*-overexpressing transgenic plants (OX2-1 and OX3-5) showing high levels of transgene expression were selected for phenotypic characterization. Hygromycin (AG Scientific, San Diego, CA, USA) resistance of the T_2 generation from these selected lines segregated as a single locus.

To generate the *bHLH34* RNA interference (RNAi) lines, the gene-specific cDNA fragments of *bHLH34* were amplified by PCR using the following primers: for *bHLH34*, forward primer 5′-GGGGACAAGTTTGTACAAAAAAGCAGGCTTCATGTATCCATCAATCGAAGACGA-3′ and reverse primer 5′-GGGGACCACTTTGTACAAGAAAGCTGGGTCCAAACTTGCTCAAGATTTCCATT-3′. The PCR products were initially cloned into the pDONR/ZEO vector and confirmed by sequencing. Subsequently, RNAi *bHLH34* cDNA constructs were directly via LR-reaction subcloned into the pB7GWIWG2(II) RNAi vector (Karimi et al., 2002) fused with the constitutive 35S promoter. The construct was then transfected into plants, and the resultant T_3 homozygous transgenic *bhlh34* RNAi lines (*ri2-2* and *ri5-1*) were used for further physiological characterization.

Statistical analysis

Statistical analyses were performed using the SPSS 23.0 software (IBM Co, Armonk, USA), including the use of one-way analysis of variance (ANOVA) and Duncan's multiple-range test. Different letters on histograms indicate that means were statistically different at the $P < 0.05$.

Supplementary References

Bechtold, N., and Pelletier, G. (1998). *In planta Agrobacterium*-mediated transformation of adult *Arabidopsis thaliana* plants by vacuum infiltration. Methods Mol. Biol. 82, 259-266. doi:10.1385/0-89603-391-0:259.

Chung, M.S., Lee, S., Min, J.H., Huang, P., Ju, H.W., and Kim, C.S. (2016). Regulation of *Arabidopsis thaliana plasma membrane glucose-responsive regulator* (*AtPGR*) expression by *A. thaliana* storekeeper-like transcription factor, AtSTKL, modulates glucose response in *Arabidopsis*. *Plant Physiol*. *Biochem*. 104, 155-164. doi:10.1016/j.plaphy.2016.03.029

Earley, K.W., Haag, J.R., Pontes, O., Opper, K., Juehne, T., Song, K., and Pikaard1, C.S. (2006). Gateway-compatible vectors for plant functional genomics and proteomics. *The Plant Journal* 45, 616-629. doi: 10.1111/j.1365-313x.2005.02617.x

Han, Y.J., Kim, Y.M., Hwang, O.J., and Kim, J.I. (2015). Characterization of a small constitutive promoter from *Arabidopsis* translationally controlled tumor protein (*AtTCTP*) gene for plant transformation. *Plant Cell Rep*. 34, 265-275. doi: 10.1007/s00299-014-1705-5.

Jefferson, R.A., Kavanagh, T.A., and Bevan, M.W. (1987). GUS fusions: *β*-glucuronidase as a sensitive and versatile gene fusion marker in higher plants. *EMBO J*. 6, 3901-3907.

Karimi, M., Inzé, D., and Depicker, A. (2002). Gateway vectors for *Agrobacterium*-mediated

plant transformation. *Trends Plant Sci*. 7, 193-195. doi: 10.1016/s1360-1385(02)02251-3

Nakagawa T, Kurose T, Hino T, Tanaka K, Kawamukai M, Niwa Y, Toyooka K, Matsuoka K, Jinbo, T., and Kimura, T. (2007). Development of series of gateway binary vectors, pGWBs, for realizing efficient construction of fusion genes for plant transformation. *J Biosci Bioeng*. 104, 34-41. doi: 10.1263/jbb.104.34

Supplementary Figures and Tables

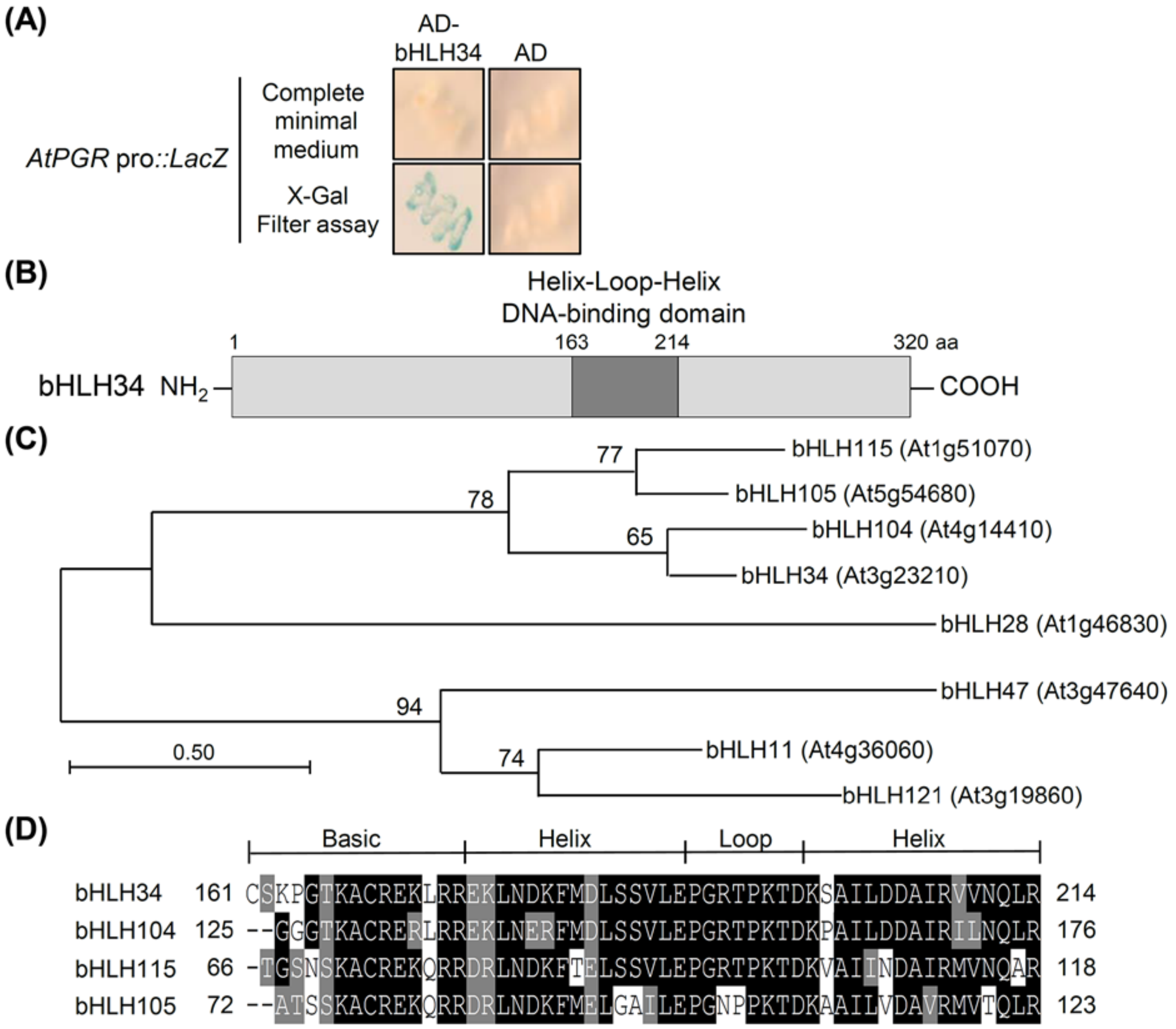

Supplementary Figure S1. Structural features and phylogenetic tree analysis of the bHLH34 protein. (A) Isolation of transcription factor candidate, bHLH34, related to *AtPGR* expression. Activation domain (AD)-bHLH34 protein-expressing yeast transformants were grown on a Leu-, Trp-, and His-free complete minimal medium. Empty vector expressing AD domain alone as negative control. *LacZ* reporter gene expression (leading to blue color on the plate containing X-gal) was driven by the *AtPGR* promoter (*AtPGR* pro::*LacZ*) in yeast. After 3 days of culturing at 30 °C, yeast colonies were picked and used for an X-Gal filter assay.

bHLH34 protein-expressing yeast colonies generated a positive color reaction within 30 min in the X-Gal filter assay. (B) The structure of the conserved region of the bHLH34 protein. The primary structure harbors a single helix-loop-helix DNA-binding site (163–214), which is indicated by the dark grey box. (C) A phylogenetic tree depicting homology relations between *Arabidopsis* bHLH34 and other bHLH members. Numbers at branch points indicate bootstrap values after 1000 replications. (D) Alignment of the basic helix-loop-helix motif of deduced amino acid sequences of the central region of bHLH34 and homologous proteins from *Arabidopsis* (bHLH104, bHLH115, and bHLH105). Black and grey shading indicates identical and similar amino acids, respectively.

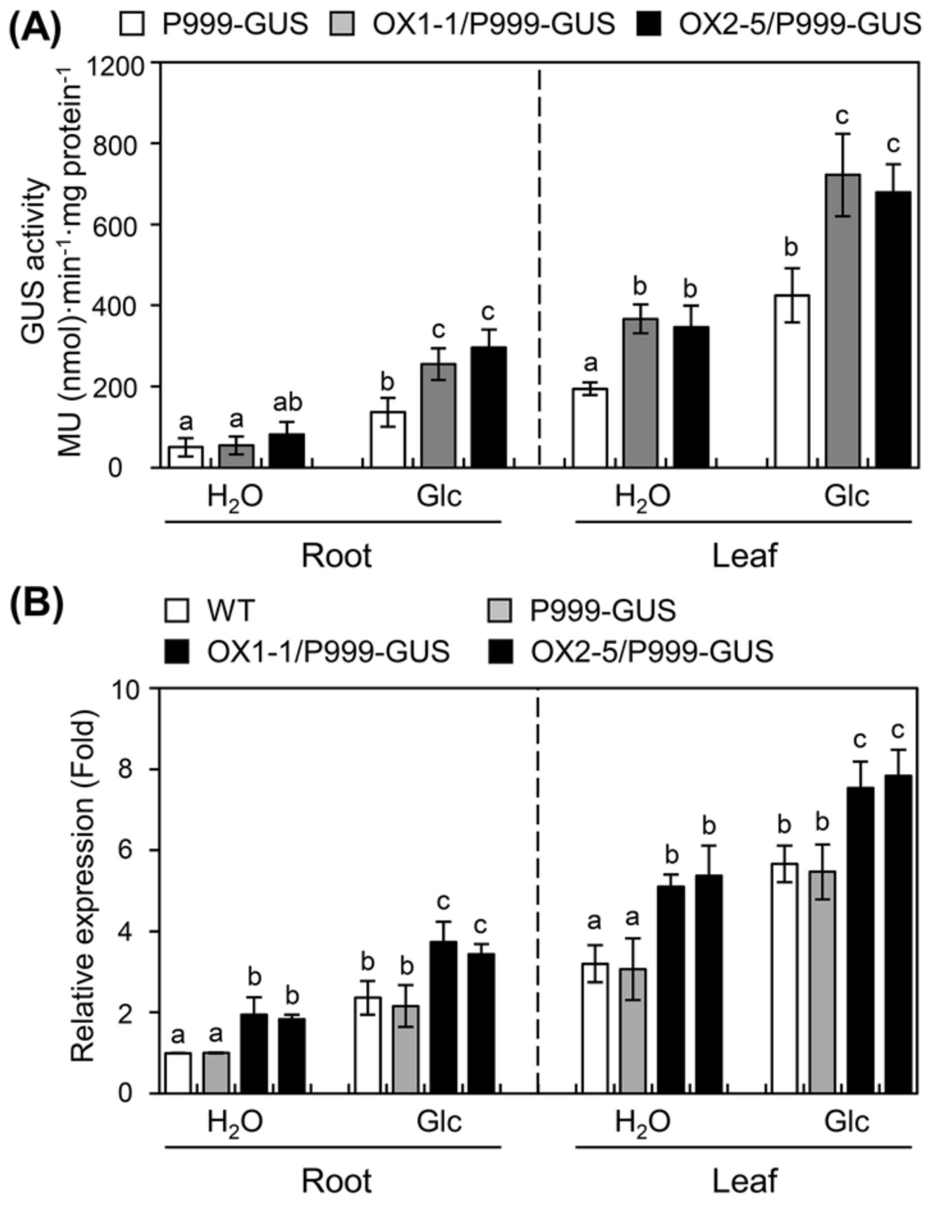

Supplementary Figure S2. GUS activities and *AtPGR* expressions in roots and leaves of P999-GUS transgenic plants under Glc treatment. (A) Average GUS activities from roots and leaves for each P999-GUS transgenic plants were calculated. These transgenic plants grown on the MS medium for 12 days were treated with H_2O or 6% glucose for 12 h. Subsequently, seedlings were subjected to GUS staining, and GUS activity was measured. The values of GUS activities are averages of three independent enzymatic assays. Each assay was performed with extracts obtained from three individual seedlings of each transgenic plant.

Error bars indicate standard deviations (ANOVA, $P < 0.05$). (B) *AtPGR* expressions were determined by qPCR using total RNA isolated from roots and leaves for WT or each P999-GUS transgenic plants. Total RNA samples were obtained from 12-day-old seedlings treated with H_2O or 6% glucose for 12 h. The mean value of three technical replicates was normalized to the level of *Actin 1* mRNA, an internal control. Error bars indicate standard deviations (n = 20 each, ANOVA, $P < 0.05$).

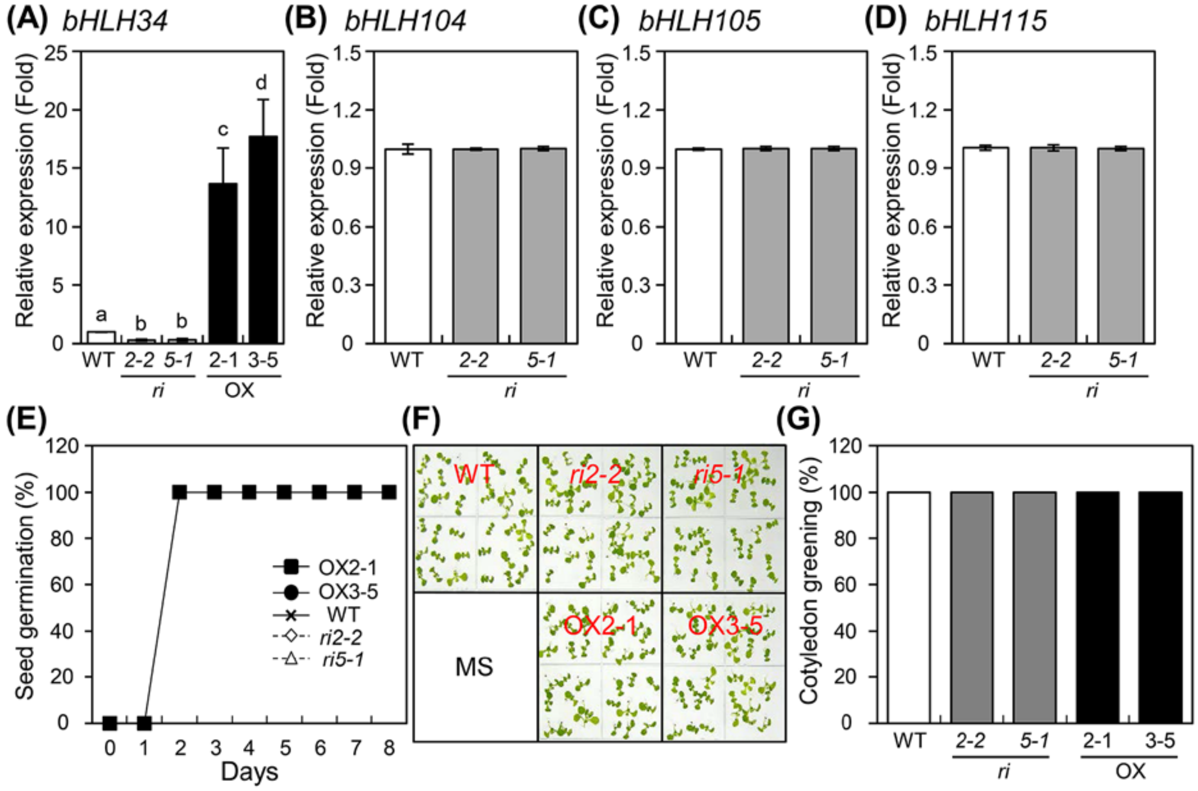

Supplementary Figure S3. Expression levels of *bHLH34* homologs and phenotypic comparison in *bHLH34* transgenic plants under the normal condition. (A) *bHLH34* expression levels in WT, *bhlh34* RNAi (*ri2-2*, *ri3-5*), and *bHLH34*-overexpressing (OX2-1, OX3-5) transgenic plants were confirmed by qPCR using RNA extracted from 14-day-old seedlings. The mean value of three technical replicates was normalized to the level of *Actin 1* mRNA as an internal control. Error bars indicate standard deviations (n = 20 each, ANOVA, P < 0.05). (B-D) *bHLH104* (B), *bHLH105* (C), and *bHLH115* (D) mRNA levels were measured by qPCR using total RNA from 14-day-old WT and two independent *bhlh34* RNAi (*ri2-2*, *ri5-1*) seedlings. The mean value of three technical replicates was normalized to the level of *Actin 1* mRNA, an internal control. Error bars indicate standard deviations (n = 20 each). *bHLH34* homologs expressions between WT and *bhlh34* RNAi plants were similar. (E) Seed germination assays in the normal condition. Seeds of WT and *bHLH34* transgenic plants were sown on the sterile MS medium and permitted to grow for indicated days, germination was scored (triplicates, n = 50 each). Error bars indicate standard deviations for three

independent experiments (50 seeds per point). WT and *bHLH34* transgenic plants seeds germinated almost evenly under normal condition. (F) Seeds of the samples were sown on the MS medium and permitted to grow for 10 days. The photograph indicates that development and green cotyledons between WT and *bHLH34* transgenic plants were similar. (G) Cotyledon greening assays in the normal condition. Seeds were sown on the MS medium and permitted to grow for 10 days, green cotyledons were counted (triplicates, n = 50 each). Error bars indicate standard deviations for three independent experiments (n = 50 seeds). There was no difference in terms of cotyledon greening rate between WT and *bHLH34* transgenic plants under the normal condition.

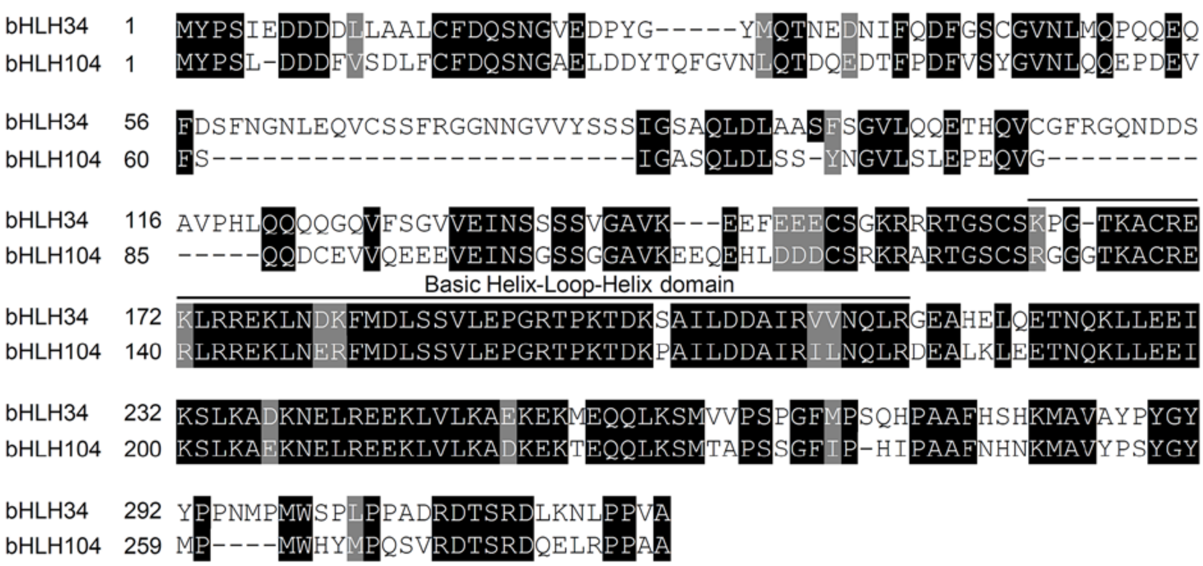

Supplementary Figure S4. Alignment of full-length deduced amino acid sequences of bHLH34 and bHLH104. Amino acid sequences of bHLH34 and bHLH104 are shown. Black and gray shading indicates identical and similar amino acids, respectively. Gaps were introduced to optimize the alignment. The basic helix-loop-helix domain is marked by a black bar above the alignment.

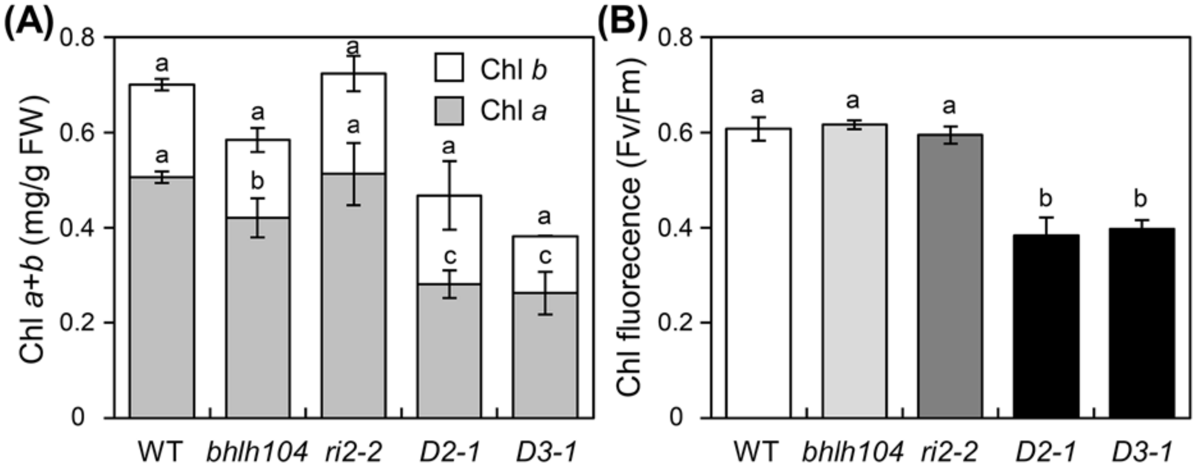

Supplementary Figure S5. Chlorophyll content and fluorescence in the *bhlh34*/*bhlh104* double mutant. (A) Quantification of total chlorophyll (Chl) *a* + *b* content in leaves of 6-day-old WT, *bhlh104*, *bhlh34* RNAi (*ri2-2*), and two independent *bhlh34*/*bhlh104* (*D2-1* and *D3-1*) plants. Values are the mean of three independent replicates of leaves of 20 mg fresh weight. Error bars represent standard deviation (ANOVA, $P < 0.05$). (B) Pale green was quantified as a decline in photochemical efficiency (Fv/Fm) of photosystem II in 6-day-old leaves of WT, *bhlh104*, *bhlh34*, and two independent *bhlh34*/*bhlh104* lines grown on the MS agar medium. Data are mean of at least 10 plants. Error bars represent standard deviation (ANOVA, $P < 0.05$).

Table S1. Primer pairs and oligonucleotides used for the electrophoresis mobility shift assay

5′-upstream region	Primer sequence (5' to 3')
P1	Forward: GAAGAAGAGACGGCCTATC<br>Reverse: TTGCTTCTTCCTTGGATCAA
P2	Forward: TGGCAGCTAGGTTCTTACAC<br>Reverse: GTGATGGAGGTGTGACGCTA
P3	Forward: TATTCGCAACCTATTCACAA<br>Reverse: ACTGGACCTGATGATCTCAT
P4	Forward: ACGGAGCTCAACGTTGGGAG<br>Reverse: AACCACGGACTCAGATCCAT
35S	Forward: CCATCATTGCGATAAAGGAA<br>Reverse: CTTTGAAGACGTGGTTGGAA
**EMSA**	**Oligonucleotide sequence**
DBE1	5'-AGAAGAGACGGC-3'<br>3'-TCTTCTCTGCCG-5'
DBE2	5'-CCAGGAGAGGTT-3'<br>3'-GGTCCTCTCCAA-5'
DBE3	5'-AAATGAGAGGAT-3'<br>3'-TTTACTCTCCTA-5'
DBE4	5'-CTTAGAGAGGAG-3'<br>3'-GAATCTCTCCTC-5'
DBE5	5'-AAATGAGATCAT-3'<br>3'-TTTACTCTAGTA-5'
DBE6	5'-GAGGGAGATTCA-3'<br>3'-CTCCCTCTAAGT-5'
M1	5'-AGAATAGACGGC-3'<br>3'-TCTTATCTGCCG-5'
M2	5'-AGAAGTGACGGC-3'<br>3'-TCTTCACTGCCG-5'
M3	5'-AGAAGATACGGC-3'<br>3'-TCTTCTATGCCG-5'
M4	5'-AGAAGAGTCGGC-3'<br>3'-TCTTCTCAGCCG-5'
M5	5'-AGAATTTTCGGA-3'<br>3'-TCTTAAAAGCCG-5'
E-box	5'-CCGGCCACTTGTGCCA-3'<br>3'-GGCCGGTGAACACGGT-5'

Table S2. Gene-specific primers used for qPCR and RT-PCR assay

Gene	Primer sequence (5' to 3')
bHLH34 (*At3g23210*)	Forward:GGGGACAAGTTTGTACAAAAAAGCAGGCTTCATGTATCCATCAATCGAAGACGA<br>Reverse:GGGGACCACTTTGTACAAGAAAGCTGGGTCAGCAACAGGAGGAAGATTTTTGA
AtPGR *(At5g19930)*	Forward: ACTGGAAGAAATGGAAACGTCGCC<br>Reverse: AGGCAGCTAAGAGTCCTGCCTT
AtHXK1 (*At4g29130*)	Forward: GACGAACCCACCAAGCTCGAG<br>Reverse: TGCATCTCAACGGTCATAGC
RAB18 *(At5g66400)*	Forward: CGATCCAGCAGCAGTATGAC<br>Reverse: TTCGAAGCTTAACGGCCACC
RD29A *(At5g52310)*	Forward: GACGGGATTTGACGGAGAAC<br>Reverse: CCGCCACATAATCTCTACCC
GIN6 *(At2g40220)*	Forward: ATGGACCCTTTAGCTTCCCA<br>Reverse: GCGAAAGTACCAAGCCACTT
AtAPR2 *(At1g62180)*	Forward: AACGCTGACTCACATTCACGAAGCG<br>Reverse: GAAAGTTCCACACATCAGCTCCTTC
ABO3 *(At1g66600)*	Forward: CCTGGAGGAAATATGGACAA<br>Reverse: CCATGAGAACAGCTTGTTCA
ABI1 *(At4g26080)*	Forward: CAAGATTCCGAGAACGGAGA<br>Reverse: GGATCAAACCGACCATCTAA
AtOZF2 *(At4g29190)*	Forward: ATGATGATCGGAGAAACTCG<br>Reverse: ACACGGCTGAGTACGGTAAC
RD29B *(At5g52300)*	Forward: CGTCCTTATGGTCATGAGC<br>Reverse: GCCTCATGTCCGTAAGAGG
bHLH104 (*At4g14410*)	Forward: ATGTATCCTTCTCTCGACG<br>Reverse: TTAAGCAGCAGGAGGCCTG
bHLH105 (*At5g54680*)	Forward: ATGGTGTCACCCGAAAACGCT<br>Reverse: CTTTAACTCTTTGATTTTGTC
bHLH115 (*At1g51070*)	Forward: ATGGTGTCTCCGGAGAATACG<br>Reverse: CTCTTCCTTGAACTTGGTTC
ACT1 (*At1g49240*)	Forward: CATCAGGAAGGACTTGTACGG<br>Reverse: GATGGACCTGACTCGTCATAC
